# Oxford Food and Activity Behaviors 20‐item questionnaire to assess personal weight management strategies: Development and testing

**DOI:** 10.1002/oby.23479

**Published:** 2022-08-03

**Authors:** Jamie Hartmann‐Boyce, Georgina Harmer, Alice Hobson, Paul A. Bateman, Kate Tudor, Paul Aveyard, Susan A. Jebb

**Affiliations:** ^1^ Nuffield Department of Primary Care Health Sciences University of Oxford Oxford UK; ^2^ Department of Psychiatry University of Oxford Oxford UK

## Abstract

**Objective:**

The aim of this study was to develop a shortened Oxford Food and Activity Behaviors (OxFAB) questionnaire to identify the cognitive and behavioral strategies used by individuals during weight‐management attempts.

**Methods:**

This study reduced an existing 117‐item questionnaire (the original OxFAB questionnaire) through identifying clusters of techniques from the responses of 278 people living with obesity and, within those clusters, identifying the most representative question or questions. Questions were rephrased to cover multiple strategies at the domain level, with several alternative phrasings developed for new questions. Face validity was tested through think‐aloud interviews with 12 people living with obesity. Questions were rephrased accordingly and tested using test‐retest (*n* = 172). Prevalence‐ and bias‐adjusted κ (PABAK) were calculated, and questions with PABAK < 0.41 were rewritten and evaluated in a new test‐retest sample (*n* = 130).

**Results:**

OxFAB20 consists of 20 questions covering diet, physical activity, and cognitive strategies for weight management. Test–retest resulted in a mean PABAK score of 0.56 (SD = 0.14). Questions were revised where appropriate. The questionnaire is available for use via a CC‐BY license.

**Conclusions:**

The OxFAB20 questionnaire provides a practical tool for researchers to identify the cognitive and behavioral strategies used by individuals during attempts at weight control.

**Study Importance**

**What is already known?**

- To optimize behavioral interventions and efforts to self‐manage weight, it is important to identify which cognitive and behavioral strategies are most effective and for whom.

**What does this study add?**

- We reduced an existing questionnaire and established its reliability and validity.

**How might these results change the direction of research?**

- The new OxFAB20 questionnaire provides a practical tool, optimized for feasible use by participants, for researchers to identify the cognitive and behavioral strategies used by individuals during attempts at weight control, and it is available free of charge.

## INTRODUCTION

Every year, most people with obesity in the United States and England attempt to lose weight; the majority do not follow formal programs [1, 2]. The year 2021 witnessed the first public health programs in England to explicitly encourage weight loss. The effect of these efforts and campaigns would increase if they could advise effective strategies. However, self‐guided weight loss has been little studied.

In 2016, we developed a complete taxonomy of the cognitive and behavioral strategies used by adults to manage their weight. We developed a questionnaire assessing the frequency with which individuals use these strategies and showed that it was reliable: the Oxford Food and Activity Behaviors (OxFAB) questionnaire [3]. Since its publication, OxFAB has been used in systematic reviews [4, 5, 6, 7, 8], as well as in primary studies [9, 10, 11]. However, a key limitation of the original questionnaire is its length; at 117 questions long, it requires a high degree of user engagement and researcher resources for analysis. When used in intervention studies, we observed high levels of noncompletion [9].

Therefore, we set out to develop a shorter version of the original OxFAB questionnaire to facilitate its wider adoption, using established methods for determining validity and reliability.

## METHODS

The University of Oxford Central University Research Ethics Committee approved this work. All participants provided informed consent. The original 117‐item questionnaire arranged strategies into domains, meaning that some strategies that were conceptually similar to each other were grouped together. In the shortened 20‐item version, we aimed to capture whether participants were using one of several strategies within a domain or set of domains. The tension we aimed to resolve was between making a question specific and clear enough while remaining open to capture all possible strategies within each domain. We used the following consecutive processes.

### Initial questionnaire reduction

We used answers from the 117‐item questionnaire to identify clusters of related questions. We used data from the following: 1) a prospective, web‐based cohort study of UK adults with overweight or obesity trying to lose weight (*N* = 486) [11]; and 2) baseline responses from a pragmatic randomized controlled trial in English adults with obesity (*N* = 278) [9]. As with the original questionnaire, multiple choice answers were coded as “yes,” including responses marked as “always” or “most of the time” or “sometimes,” and “no,” including responses marked as “never” or “hardly ever” or “not relevant to me.” First, we removed four questions for which more than 85% of respondents in both data sets indicated use of the strategy, because these strategies were considered to be core and unlikely to contribute to meaningful analyses. We then used cluster analysis to identify groups of at least two questions for which answers were similar in both data sets and identified questions around which the clusters centered. Using these data, we developed representative questions, based on the centered question, covering questions in the cluster. In some cases, and particularly for larger clusters, we drafted multiple questions per cluster. We also considered the theoretical domains from the original questionnaire in order to remain faithful to the original taxonomy.

### Think aloud (cognitive testing)

Think aloud is a form of cognitive interviewing designed to provide verbal data about reasoning during set tasks [12]. It is often used to establish validity as part of questionnaire development [13, 14]. A total of 12 participants were purposively sampled from the general public with a range in socioeconomic status and a gender balance, using social media (e.g., Facebook), email circulation from previous research in this area, and snowball sampling. We included UK adults (age ≥18 years) who are fluent English speakers living with overweight/obesity and trying to lose weight or maintain weight loss through changing diet and/or physical activity. Via telephone interviews, the interviewer read out each question and asked participants to answer the question while talking through their reasoning. When there were multiple questions for a single strategy, participants were asked to indicate which they preferred and why. Interviews were audiotaped, with key quotes transcribed by one researcher and reviewed by a team of four. When participants raised concern or uncertainty or when reasons given for responses were not congruent with question intent, questions were rephrased. When multiple questions existed for one strategy, we selected the question with responses most congruent with the question's intent.

### Test–retest

We assessed reliability using web‐based test–retest surveys. Participants were required to repeat the questionnaire 1 to 2 weeks after initial completion.

Participants were recruited through the community using email circulation lists, social media, snowball sampling, research recruitment sites, and existing department contacts and volunteer databases. Inclusion criteria were as described earlier.

For the initial test‐retest round, target sample size was 130, based on a calculation of 126 to achieve 80% power to detect a prevalence‐ and bias‐adjusted κ (PABAK) of at least 0.41 (considered moderate agreement) [15]. This threshold was chosen because some genuine changes in behavior were expected between the two response dates.

Multiple choice answers were coded as “yes” or “no” as per initial questionnaire reduction. Using data from the two testing rounds, the PABAK was calculated for each question [16]. Questions for which test‐retest resulted in PABAK scores <0.41 were reevaluated and rephrased as appropriate and then tested again in a new sample of 130 participants, meeting the same inclusion criteria as the first round [17]. Using only the responses to the initial questionnaire to avoid double counting, we also intended to rephrase and retest questions with more than four participants indicating “unclear;” no questions fit this criterion.

## RESULTS

### Initial questionnaire reduction

Cluster analysis resulted in 11 clusters. Through discussion, we developed from this a list of 21 questions covering all clusters as well as relevant theoretical domains not clearly identified from clusters. For some questions, more than one wording was tested.

### Think‐aloud testing

Twelve participants were interviewed, reflecting men and women, a range of ages, and different educational backgrounds. Saturation was judged to have been reached when multiple interviewees were repeating answers from previous interviews. Think aloud led to the removal of some questions, the combining of two questions, and amending of others. Amendments included changes to wording and adding in specific examples of a representative behavior. This resulted in a list of 20 revised questions.

### Test–retest

#### Round one

The first survey was completed by 172 participants (Table 1), of which 134 completed the second survey. There were no significant differences in characteristics between those who completed both rounds and those who completed baseline only. At baseline, the mean number of strategies used was 13 (SD = 4).

**TABLE 1:** Participant demographics, test‐retest rounds 1 (*n* = 172) and 2 (*n* = 130)

	Round 1 (*N* = 172)		Round 2 (*N* = 130)	
	*n*	%	*n*	%
Female	106	62	76	58
Ethnicity				
African	7	4	35	27
Any mixed/multiple ethic background	10	6	8	6
Any other ethnic group	5	3	10	9
Any other White background	68	40	50	38
White British	70	41	24	18
Indian	12	7	3	2
Education				
None	2	1	1	1
GCSE or equivalent	11	6	12	9
A levels or equivalent	37	22	33	25
University undergraduate degree	65	38	51	39
University postgraduate degree	53	31	27	21
Prefer not to say	4	2	6	5
Age				
18‐24	52	30	39	30
25‐29	27	16	33	25
30‐34	21	12	25	19
35‐39	18	10	18	14
40‐44	14	8	8	6
45‐49	14	8	4	3
50‐59	16	9	1	1
60+	10	6	2	2

Abbreviation: GCSE, General Certificate of Secondary Education.

Results for each question can be seen in Table 2. The mean PABAK score was 0.56 (SD = 0.14). Three questions (questions 4, 8, and 19) had PABAK < 0.41. We drafted alternate wording for these questions and then tested them again using test‐retest (round 2).

**TABLE 2:** Results from reliability testing

No.	Question	Round 1			Round 2		
		Percent using (*n* = 172)	*n* unclear (*n* = 172)	PABAK (*n* = 134)	Question	*n* unclear (*n* = 130)	PABAK (*n* = 130)
**1**	I have specific goals to help me lose weight (e.g., a weight‐loss goal, running 5 k, eating 5 servings of fruit or vegetables a day)	80%	1	0.65	n/a		
**2**	I have a detailed plan of what and when I'm going to eat or drink or the exercise I am going to do as a way to help me lose weight	66%	2	0.5	n/a		
**3**	I go to a weight‐loss group or program or have recently talked to a professional about losing weight	26%	3	0.71	n/a		
**4**	I try to get other people (e.g., friends, family, social media, or colleagues) to support me in losing weight	52%	1	0.28	(a) I try to get other people (e.g., friends, family, social media, colleagues) to support me in losing weight	0	0.56
					(b) I have asked other people (e.g., friends, family, social media, colleagues) to support me in losing weight	0	0.56
**5**	I use weight‐loss aids such as apps, equipment, or diet foods to help me lose weight	70%	0	0.67	n/a		
**6**	I try to balance my energy intake and how much energy I use (e.g., allowing myself a biscuit if I go to the gym, eating a small meal in the day if I'm going out for dinner, going on a long run if I've been inactive)	73%	1	0.47	n/a		
**7**	I check how my weight loss is going (e.g., weighing myself regularly, checking how my clothes fit, recording what I eat or the exercise/steps I do)	89%	0	0.75	n/a		
**8**	If I want to do something that does not fit in with my weight‐loss plans, like eat something outside of my diet plan or not exercise, I ask myself why I feel that way	52%	2	0.27	(a) If I want to do something that does not fit in with my weight‐loss plans, like eat something outside of my diet plan or not exercise, I ask myself why I feel that way	0	0.35
					(b) If I want to eat something outside of my diet plan or not exercise, I ask myself why I feel like this	0	0.35
					(c) If I want to do something that does not fit with my plans, I ask myself why I feel that way; for example, am I really hungry?	0	0.44
**9**	There are things I do to help me avoid or resist temptation	77%	0	0.71	n/a		
**10**	I have ways to boost my motivation to lose weight (e.g., reminding myself about why I want to lose weight, rewarding myself if I lose weight)	81%	0	0.64	n/a		
**11**	If I'm eating out, I think ahead about what I'm going to eat and drink or how I'm going to turn down food if people offer it to me	55%	1	0.46	n/a		
**12**	When food shopping, I have ways to help me buy foods that fit with my weight‐loss plans (e.g., use a shopping list, do not shop when I'm hungry, avoid certain aisles, shop online)	73%	0	0.61	n/a		
**13**	I am trying to lose weight alongside one or more people (e.g., friend/family member/partner)	41%	0	0.53	n/a		
**14**	I have a plan for losing weight, but I allow myself to be flexible about what I do depending on circumstances	80%	0	0.67	n/a		
**15**	I have ways to remind myself to exercise	65%	2	0.48	n/a		
**16**	When I'm feeling hungry or if I am uncomfortable when exercising, I acknowledge and accept the feeling	83%	2	0.71	n/a		
**17**	I avoid certain foods or drinks or certain situations as a way to help me stick to my weight‐loss plans	83%	0	0.64	n/a		
**18**	I've made changes to my surroundings to help me lose weight (e.g., using smaller plates or bowls, keeping certain foods out of the house)	64%	0	0.63	n/a		
**19**	My weight‐loss strategy allows me to eat as much as I want of certain types of food and drinks	55%	2	0.32	(a) My weight‐loss strategy allows me to eat as much as I want of certain types of food and drinks	1	0.37
					(b) My weight‐loss strategy includes some unlimited foods (e.g., “free foods”)	1	0.34
**20**	I do not think of myself as on a diet. Instead, I think about this as a new way of life, so I feel positive about what I am doing	72%	1	0.48	n/a		

Abbreviation: PABAK, prevalence‐ and bias‐adjusted κ.

#### Round two

The three questions were rephrased into multiple versions (Table 2). The sample for round 2 (*n* = 130) was similar to that in round 1 (Table 1), with the exception of a higher proportion of participants reporting African and any other White ethnicity and a smaller proportion identifying as White British.

Both options for question 4 had PABAK values above the threshold; we selected option (b), as that had the highest kappa. For question 8, we retained option (c) as the only one with PABAK > 0.41. For question 19, neither option had PABAK of >0.41. We retained option (a), as it had the higher of the two, and alternative improved phrasings could not be identified that remained faithful to the taxonomy.

The final questionnaire is in Table 3. The response options tested were unchanged from those validated for the original 117‐item questionnaire.

**TABLE 3:** OxFAB20 questionnaire

No.	Question	Domain
**1**	I have specific goals to help me lose weight (e.g., a weight‐loss goal, running 5 k, eating 5 servings of fruit or vegetables a day)	Goal setting
**2**	I have a detailed plan of what and when I'm going to eat or drink or the exercise I am going to do as a way to help me lose weight	Planning content; scheduling of diet and activity
**3**	I go to a weight‐loss group or program or have recently talked to a professional about losing weight	Support: professional
**4**	I have asked other people (e.g., friends, family, social media, colleagues) to support me in losing weight	Support: motivational
**5**	I use weight‐loss aids such as apps, equipment, or diet foods to help me lose weight	Weight‐management aids
**6**	I try to balance my energy intake and how much energy I use (e.g., allowing myself a biscuit if I go to the gym, eating a small meal in the day if I'm going out for dinner, going on a long run if I've been inactive)	Energy compensation
**7**	I check how my weight loss is going (e.g., weighing myself regularly, checking how my clothes fit, recording what I eat or the exercise/steps I do)	Self‐monitoring
**8**	If I want to do something that does not fit with my plans, I ask myself why I feel that way; for example, am I really hungry?	Impulse management: awareness of motives
**9**	There are things I do to help me avoid or resist temptation	Impulse management: awareness of motives; impulse management: distraction; impulse management: acceptance; stimulus control
**10**	I have ways to boost my motivation to lose weight (e.g., reminding myself about why I want to lose weight, rewarding myself if I lose weight)	Motivation
**11**	If I'm eating out, I think ahead about what I'm going to eat and drink or how I'm going to turn down food if people offer it to me	Planning content; regulation: rule setting
**12**	When food shopping, I have ways to help me buy foods that fit with my weight‐loss plans (e.g., use a shopping list, do not shop when I'm hungry, avoid certain aisles, shop online)	Planning content; stimulus control; regulation: rule setting; regulation: restrictions
**13**	I am trying to lose weight alongside one or more people (e.g., friend/family member/partner)	Support: buddying
**14**	I have a plan for losing weight, but I allow myself to be flexible about what I do depending on circumstances	Regulation: restraint (flexible restraint)
**15**	I have ways to remind myself to exercise	Stimulus control
**16**	When I'm feeling hungry or if I am uncomfortable when exercising, I acknowledge and accept the feeling	Impulse management: acceptance
**17**	I avoid certain foods or drinks or certain situations as a way to help me stick to my weight‐loss plans	Regulation: restrictions
**18**	I've made changes to my surroundings to help me lose weight (e.g., using smaller plates or bowls, keeping certain foods out of the house)	Stimulus control
**19**	My weight‐loss strategy allows me to eat as much as I want of certain types of food and drinks	Regulation: allowances
**20**	I do not think of myself as on a diet. Instead, I think about this as a new way of life, so I feel positive about what I am doing	Reframing

Abbreviation: OxFAB, Oxford Food and Activity Behaviors.

## DISCUSSION

We have developed and tested a short 20‐item questionnaire, based on the previously established 117‐item OxFAB questionnaire, to record the behavioral strategies used by individuals to manage their weight and established its reliability and face validity. This can now be adopted into future research studies. Our sample included a range of ethnicities, educational backgrounds, and weight‐loss experiences, but online recruitment may have excluded some groups of participants, and further work may need to be done to test its application to other samples.

The longer OxFAB questionnaire [3] has already been used in intervention and observational studies [9, 10, 11]. However, researchers have asked for a shorter questionnaire. The 20‐item version is not simply a shorter version of the 117‐item questionnaire. The 117‐item questionnaire measures strategies being used, and thereby domains, whereas the 20‐item version assesses whether one of several strategies are being used but does not aim to capture which strategy that is. Each question in the 20‐item version maps on to one or more domains (Table 3), with 19 domains covered in total. More information on the significance and background to the domains can be found in Hartmann‐Boyce 2016 [3].

The OxFAB questionnaire is, to our knowledge, unique in aiming to quantify the behaviors enacted by individuals to manage their weight. Other questionnaires seek to quantify energy intake and expenditure [18, 19] or actions by therapists [20]. We developed OxFAB to capture self‐guided weight‐loss attempts, but it could be used by therapists to support individuals or to incorporate into programs to enhance their effectiveness.

Our new, short questionnaire (OxFAB20) is available to use free of charge via a CC‐BY license, which we hope will enable researchers, commissioners, and the public to gain a better understanding of the cognitive and behavioral strategies most closely linked with successful weight loss.

## FUNDING INFORMATION

We are grateful to the National Institute for Health Research (NIHR) Oxford Biomedical Research Centre (BRC) Obesity, Diet, and Lifestyle Theme and NIHR Oxford Applied Research Centre for providing funding for this work. Paul Aveyard and Susan A. Jebb are funded by NIHR Oxford Applied Research Centre. The views expressed are those of the authors and not necessarily those of the National Health Service, the NIHR, or the Department of Health and Social Care.

## CONFLICT OF INTEREST

The authors declared no conflict of interest.
